# Effects of Dietary Carbohydrate to Lipid Ratios on Growth Performance, Muscle Fatty Acid Composition, and Intermediary Metabolism in Juvenile Black Seabream (*Acanthopagrus schlegelii*)

**DOI:** 10.3389/fphys.2020.00507

**Published:** 2020-06-03

**Authors:** Sehrish Taj, Misbah Irm, Min Jin, Ye Yuan, Hardy Joël Timothée Andriamialinirina, Qicun Zhou

**Affiliations:** Laboratory of Fish and Shellfish Nutrition, School of Marine Sciences, Ningbo University, Ningbo, China

**Keywords:** black seabream, CHO:L ratios, glycolysis, gluconeogenesis, lipogenesis, long-chain PUFA

## Abstract

An 8-week feeding trial was conducted to evaluate the effects of dietary carbohydrate to lipid (CHO:L) ratios on growth performance, muscle fatty acid composition, and intermediary metabolism in juvenile black seabream (*Acanthopagrus schlegelii*). Five isonitrogenous and isoenergetic diets (48.0% crude protein and 18.0 MJ kg^–1^ gross energy) were formulated to contain different CHO:L ratios ranging from 0.33 to 3.75. Triplicate groups of 20 fish averaging 0.51 ± 0.01 g were fed with experimental diets twice daily to apparent satiation. The results indicated that final body weight (FBW), percentage weight gain (PWG), specific growth rate (SGR), and protein efficiency ratio (PER) were significantly influenced by the dietary CHO:L ratios (*p* < 0.05). The highest FBW, PWG, and SGR were observed in fish fed the diet with a CHO:L ratio of 1.36 (*p* < 0.05). A two-slope broken-line regression analysis based on PWG indicated that the optimal dietary CHO:L is 1.08. Lipid content in the whole body decreased, and glycogen concentration in the liver increased with the increase of dietary CHO:L ratios from 0.33 to 3.75 (*p* < 0.05). Moreover, there was a positive correlation between muscle fatty acid composition and dietary fatty acid composition. The relative expression levels of genes involved in glucose metabolism, such as *gk*, *pepck*, and *glut2* were upregulated by increasing the dietary CHO:L ratio. Also, the mRNA expression level of genes related to lipid synthesis, such as *fas* and *acc*α were significantly upregulated with dietary CHO:L ratios increasing from 0.33 to 3.75. The highest expression of genes involved in fatty acid β-oxidation, such as *cpt1* and *acox1*, were observed in fish fed the 1.36 CHO:L ratio diet. The gene expression of Δ6 fatty acyl desaturase (*fads2*) in the liver significantly increased with increase of dietary CHO:L ratios from 0.33 to 3.75. Fish fed the diet with CHO:L ratios of 2.26 and 3.75 had lower expression levels of *elovl5* than those fed the other diets. These results demonstrate that dietary optimal CHO:L ratios could improve PWG and SGR but also influence expression of genes involved in glucose and lipid metabolism. Based on the overall results, the optimal dietary CHO:L ratio is 1.08 for black seabream.

## Introduction

Development of the cost-effective and nutritionally adequate formulated diet is fundamental to the future feasibility for marine fish culture. The rapid growth of aquaculture output depends on the production of aquatic feed, whereas the supply of fish meal and fish oil, which are the most important ingredients in aqua-feeds, has remained comparatively static and gradually decreased over the last decade (Oliva-Teles et al., 2015; Matulić et al., 2020; Wang et al., 2020). In aquaculture, aqua-feed cost are very high, accounting for more than 60% of the total production cost. The fish meal is known as the best protein for aqua-feeds (Han et al., 2016; Hua et al., 2019). Fish meal resources are limited, and fish meal prices have recently increased as populations of wild fisheries have declined due to overfishing (Hutchins et al., 1998; Hardy, 2010; Raggi et al., 2019; Ma et al., 2020). To minimize dietary protein levels, much attention was given to analyzing the viability of non-protein energy substitutes and proved that providing sufficient energy with dietary lipids and carbohydrates can reduce the use of costly protein (Kim and Lee, 2005; Li et al., 2012a, b; Darias et al., 2015). Therefore, dietary carbohydrates and lipids are of increasing importance to aquaculture as these two main nutrients are used as non-protein energy sources in fish feeds and reduce protein requirements (Lin and Shiau, 2003; Li et al., 2012a, b, 2019).

Carbohydrates and lipids are cheaper sources of energy compared to proteins, and they can spare dietary protein for growth rather than being used as an energy source, which is also coupled with increased ammonia excretion into the water. Fish growth performance and metabolic efficiency vary depending on the dietary lipid and carbohydrate levels (Gümüş and Ikiz, 2009; Li et al., 2014; Xing et al., 2016). Any imbalances in the supply of lipids and carbohydrates negatively affect growth performance, nutrient utilization, and even the health status of fish (Erfanullah and Jafri, 1998; Li et al., 2012b). Recently, an increasing number of studies have investigated the interactions between lipid and carbohydrate levels in fish species, such as red drum (*Sciaenops ocellatus*; Ellis and Reigh, 1991), rainbow trout (*Oncorhynchus mykiss*; Gümüş and Ikiz, 2009), blunt snout bream (*Megalobrama amblycephala*; Li et al., 2014), and large yellow croaker (*Larmichthys crocea*; Zhou et al., 2016). However, most studies mainly focused on determining the most favorable carbohydrate-to-lipid ratio for growth performance (Gao et al., 2010; Miao et al., 2016). The effects of different dietary CHO:L ratios on the molecular glucose and lipid metabolism in fish have rarely been investigated.

There is a strong interaction between glucose and fatty acids in fish, and this affects glucose and lipid utilization (Menoyo et al., 2006; Song et al., 2018). Dietary lipids can be converted to glucose through gluconeogenesis, and alternatively, glucose can be deposited as lipids in fish tissue (Honorato et al., 2010). The dietary lipid and carbohydrate levels regulate relative expression of genes involved in glucose and lipid metabolism. The dietary carbohydrate or glucose administration was noted to enhance lipogenesis (Kamalam et al., 2012). On the contrary, the β-oxidation data are conflicting as either stimulation (Kamalam et al., 2013) or inhibition (Jin et al., 2014) effects have been reported. Regarding long-chain polyunsaturated fatty acid (LC-PUFA) biosynthesis, the expression of induction of desaturase and elongase were also reported in freshwater fish and salmonids (Seiliez et al., 2001). But in marine fish, such induction of genes was not clear (González-Rovira et al., 2009; Vagner and Santigosa, 2011). In salmonids, desaturases and elongases were upregulated by dietary carbohydrates (Seiliez et al., 2001; Kamalam et al., 2013), but in marine fish species, such an effect has never been confirmed (Castro et al., 2015). These studies suggest that the growth performance and intermediary metabolism of fish might be affected by the interaction between dietary CHO:L ratios, which were seldom evaluated in marine fish; thus, special attention is required in carbohydrate or lipid studies.

Black seabream (*Acanthopagrus schlegelii*) is a popular and commercially important marine carnivorous fish species cultured in China, Japan, Korea, and some countries of Southeast Asia due to its high economic value (Nip et al., 2003; Jin et al., 2017). This species is a good candidate for intensive culture because it has many desirable characteristics, such as resistance to disease, rapid growth rate, good meat quality, and ability to tolerate environmental changes (Hong and Zhang, 2003; Shao et al., 2008; Kalhoro et al., 2018). However, the feed used for black seabream farming are trash fish traditionally, and they do not meet the nutrient requirements to sustain optimum growth and cause water pollution (Ma et al., 2008; Jin et al., 2017). The nutritional studies demonstrate that black seabream need lipid and protein in diets, approximately 14 and 40%, respectively (Shao et al., 2008; Peng et al., 2009; Zhang et al., 2010). Currently, there is no report available regarding the optimal dietary carbohydrate-to-lipid ratio requirement and glucose utilization in black seabream. The objective of the present study was to evaluate the effects of dietary CHO:L ratio on growth performance, muscle fatty acid composition, and intermediary metabolism in juvenile black seabream. Furthermore, the results obtained from this study might present some new insight into the non-protein energy utilization by fish and might facilitate the advancement of the low-protein and high-energy feed for black seabream.

## Materials and Methods

### Diets Preparation

All feed ingredients were purchased from Ningbo Tech-Bank Corp., Ningbo, China. The formulation and proximate composition of the experimental diet are presented in Table 1. Five isonitrogenous and isoenergetic diets (48.0% crude protein and 18.0 MJ kg^–1^ gross energy) were formulated to contain various CHO:L ratios ranging from 0.33 to 3.75. Fish meal and soybean meal were used as protein sources. The soybean oil and fish oil in equal amounts were used as the lipid sources, and dextrin was used as the carbohydrate source. Cellulose was used to equilibrate the carbohydrate levels required. All dry ingredients were ground into fine powder with a particle size less than 177 microns and micro-components, such as minerals and vitamin premix, were added, followed by an appropriate quantity of oil and water (35% w/w). The ground ingredients were mixed in a Hobart-type mixer until homogenous, and cold-extruded pellets were produced using a twin screws extruder (F-26, machine factory of South China University of Technology, Guangzhou, China). The pellet strands were cut off into two uniform sizes of 2 and 4 mm in diameter using a granulating machine (G-250, Machine factory of South China University of Technology, Guangzhou, China). Pellets were steamed for 30 min at 90°C. Then pellets were air-dried to approximately 10% moisture. All diets were stored at −20°C in plastic-lined bags until use in the feeding trial. The fatty acid profiles of the experimental diets were determined with few modifications (Zuo et al., 2013; Jin et al., 2017). Fatty acid methyl esters were separated and measured by GC-MS (Agilent technologies 7890B-5977A). Results are presented as a percentage of total fatty acids in Table 2.

**TABLE 1:** Formulation and proximate composition of experimental diets (% dry matter).

**Ingredients (%)**	**Dietary carbohydrate to lipid ratios**				
	**0.33**	**0.76**	**1.36**	**2.26**	**3.75**
Fish meal^a^	40	40	40	40	40
Soybean meal^a^	24	24	24	24	24
Dextrin^a^	6	12	18	24	30
Fish oil^a^	6.5	4.2	2.9	1.6	0.3
Soybean oil^a^	6.5	4.2	2.9	1.6	0.3
Soybean lecithin^a^	1.9	1.9	1.9	1.9	1.9
Vitamin supplement^b^	0.5	0.5	0.5	0.5	0.5
Mineral supplement^c^	1.0	1.0	1.0	1.0	1.0
Ca(H_2_PO_4_)_2_	1.7	1.7	1.7	1.7	1.7
Choline chloride	0.3	0.3	0.3	0.3	0.3
Cellulose	11.6	10.2	6.8	3.4	0
**Proximate composition (%)**					
Moisture	9.88	10.18	9.57	9.24	10.19
Crude protein	47.02	47.13	47.14	48.97	48.44
Crude lipid	15.41	13.14	11.22	7.95	6.48
Ash	6.68	6.45	6.58	6.92	5.56
Energy (MJ⋅kg^–1^)^d^	18.06	18.05	18.05	18.06	18.06
Crude fiber	15.91	13.05	10.18	8.92	5.03
Carbohydrate: lipid (CHO:L)	0.33	0.76	1.36	2.26	3.75
Nitrogen-free extract^e^	5.10	10.05	15.31	18.00	24.30

*^*a*^Ningbo Tech-Bank Feed Co., Ltd., China. ^*b,c*^Similar as (Jin et al., 2017). ^*d*^Calculated values based on 23.6, 39.5, and 17.2 kJ/g for protein, lipid and carbohydrate respectively. ^*e*^Nitrogen-free extract content = 100 – moisture – crude protein – crude lipid – ash – crude fiber.*

**TABLE 2:** Fatty acid composition of experimental diets (% total fatty acids).

**Parameters**	**Dietary carbohydrate to lipid ratios**				
	**0.33**	**0.76**	**1.36**	**2.26**	**3.75**
C14:0	3.85	3.84	3.84	3.80	3.85
C16:0	17.65	17.84	17.84	18.21	18.82
C18:0	5.15	5.12	5.12	5.08	4.97
C20:0	0.45	0.40	0.03	0.43	0.37
SFA^1^	27.10	27.19	26.83	27.52	28.01
C16:1n	3.89	3.72	3.62	3.42	3.20
C18:1n-9	16.69	15.83	14.95	13.97	11.44
C20:1n-9	1.82	1.69	1.62	1.47	1.33
C22:1n-11	0.37	0.28	1.67	0.25	0.15
MUFA^2^	22.78	21.53	21.86	19.11	16.12
C18:2n-6	24.67	23.95	22.76	21.67	18.88
C18:3n-6	0.09	0.08	0.12	0.09	0.08
C20:2n-6	0.16	0.16	0.14	0.16	0.19
C20:4n-6	0.52	0.53	0.54	0.52	0.56
C22:4n-6	0.05	0.07	0.07	0.09	0.07
n-6 PUFA^3^	25.49	24.79	23.62	22.52	19.79
C18:3n-3	3.25	3.18	3.10	2.94	2.62
C18:4n-3	1.06	1.07	1.10	1.11	1.17
C20:4n3	0.47	0.32	0.33	0.34	0.36
C20:5n-3 (EPA)	4.96	4.80	4.81	4.90	5.02
C22:5n-3 (DPA)	0.68	0.72	0.74	0.76	0.76
C22:6n-3 (DHA)	7.41	7.52	7.88	8.61	9.57
n-3 PUFA^4^	17.84	17.62	17.96	18.65	19.50
EPA + DHA	12.37	12.33	12.70	13.51	14.59

*Data are presented as means ± SE (*n* = 3). Some fatty acids, found in only trace amounts or not detected, such as C8:0, C12:0, C13:0, C15:0, C14:1n-7, and C20:5n-6 were not listed in Table 2. ^1^SFA; saturated fatty acids; ^2^MUFA, mono-unsaturated fatty acids; ^3^n-6 PUFA; n-6 polyunsaturated fatty acids; ^4^n-3 PUFA; n-3 polyunsaturated fatty acid.*

### Feeding Trial

The juvenile black seabream were purchased from a local commercial hatchery at Xiangshan Bay, Ningbo, China. All fish were acclimated for 2 weeks prior to experimentation and were fed a commercial diet (Ningbo Tech-Bank Corp, Zhejiang, China; 45% crude protein and 12% lipid) as described previously (Jin et al., 2017). At the beginning of the experiment, fish were fasted for 24 h. Then, a total of 300 juvenile black seabream of almost similar size (initial weight 0.51 ± 0.01 g) were randomly distributed into 15 300-L cylindrical fiberglass tanks filled with 250 L of water at the stocking rate of 20 fish per tank. Each experimental diet was randomly assigned to three replicates. During the feeding trial, fish were fed with experimental diets twice a day (08:00 and 17:00) to apparent satiation. All tanks were provided with a continuous flow of water (0.5 L min^–1^) and water was continuously aerated with air stones to maintain the dissolved oxygen level near saturation. During the experimental period, water temperature was 27–33°C, pH was 6.7–7.7, salinity was 22–26 mg L^–1^, ammonia nitrogen was lower than 0.05 mg L^–1^, and dissolved oxygen content was 6.5–7.6 mg L^–1^, and all were measured daily with a YSI Pro plus instrument (YSI, Yellow Springs, OH, United States). The experimental units were under a natural light and dark cycle.

### Sample Collection

In the present study, all procedures complied with Chinese law pertaining to experimental animals. The protocol was approved by the Ethic-Scientific Committee for Experiments on Animals of Ningbo University. At the end of the feeding trial, fish in each tank were sampled 24 h after the last feeding. The fish in each tank were anesthetized with MS-222 (Shanghai Reagent Corp., Shanghai, China) and then individually weighed, counted, and sampled to determine survival, percentage weight gain (PWG), specific growth rate (SGR), feed conversion ratio (FCR), and protein efficiency ratio (PER). Five fish from each tank were randomly sampled and frozen at −20°C to analyze whole-body proximate composition. Hepatosomatic index (HSI), viscerosomatic index (VSI), and condition factor (CF) were determined from three individual fish per tank by obtaining tissues (livers and viscera) and expressing ratios as a percentage of body weight. Muscle samples were also collected from three fish per tank to analyze the fatty acid composition. Blood was sampled from the caudal vasculature of five fish per tank using 1 ml heparinized syringes and stored at 4°C. Then the blood samples were centrifuged at 956 × *g* for 10 min at 4°C to separate the serum for biochemical indices analysis. The liver from five fish after taking a blood sample in each tank were pooled into 1.5 ml eppendorf tubes and immediately frozen in liquid nitrogen and then stored at −80°C for gene expression analysis.

### Proximate Composition Analysis

Proximate composition of whole body and diets were analyzed following the standard procedures of the Association of Official Analytical Chemists (AOAC, 2006). Moisture content was determined by drying the samples to a constant weight at 105°C. Crude protein (*N* × 6.25) was determined via the Dumas combustion method with a protein analyzer (Leco FP528, St. Joseph, MI, United States). The crude lipid was determined by the ether extraction method using the Soxhlet Method (Soxtec System HT6, Tecator, Sweden), and ash content was determined by using a muffle furnace at 550°C for 8 h. The crude fiber was analyzed by the fritted glass crucible method using an automatic analyzer (ANKOM A2000i, Macedon, New York, NY, United States).

### Serum Biochemical Analysis

The serum biochemical parameters, including glucose (GLU), triglyceride (TG), cholesterol (CHOL), and total protein (TP), were measured by an automatic biochemical blood analyzer (Selectra Pro-M 13-7476). The glycogen contents in liver were determined by the assay kit (No. A043; Jian Cheng Bioengineering Institute, Nanjing, China) as previously described (Hassid and Abraham, 1957).

### Fatty Acid Analysis

The fatty acid profiles of experimental diets and fish muscle tissue were determined with a few modifications (Zuo et al., 2013). The freeze-dried samples were added to 12-ml volumetric glass screw cap tubes (Teflon gasket), 3 ml potassium hydroxide in methanol (1 N) was added and heated at 72°C in a water bath for 20 min. After cooling, 3 ml of 2 N HCl in methanol was added and the mixture heated at 72°C in a water bath for 20 min. Finally, 1 ml hexane was added to the mixture, shaken vigorously for 1 min, and then permitted to separate into two layers. Fatty acid methyl esters were separated and identified by GC-MS (Agilent technologies 7890B -5977A) as previously detailed (Jin et al., 2017). Results are presented as a percentage of total fatty acids.

### Total RNA Extraction, Reverse Transcription and Real-Time PCR

Total RNA was extracted from the liver tissues using TRIzol reagent (Takara, Japan) according to the manufacturer’s instructions. Quantity and quality of isolated RNA were determined spectrophotometrically (Nanodrop 2000, Thermo Fisher Scientific) and on a 1.2% denaturing agarose gel, respectively. The cDNA was generated from 1,000 ng of DNase-treated RNA and synthesized by a Prime Script^TM^ RT Reagent Kit with gDNA Eraser (perfect Realtime; Takara, Japan). The housekeeping gene β*-actin* was used as reference gene after confirming its stability across the experimental treatment. Specific primers for the candidate genes glucokinase (*gk*), pyruvate kinase (*pk*), phosphoenolpyruvate (*pepck*), glucose-6-phosphatase (*g6pc*), glucose transporter 2 (*glut2*), acetyl-coA carboxylase alpha (*acc*α), fatty acid synthase (*fas*), carnitine palmitoyl transferase 1 (*cpt1*), acyl-COA oxidase (*acox1*), fatty acyl desaturase (*fads2*), and elongase 5 (*elovl5*) used for qPCR were designed using Premier 3.0 software (Table 3). The primer specificities of the candidate genes were checked as previously detailed (Bustin et al., 2010) by systematically running melting curve assays after the qPCR program and DNA sequencing technology (BGI, China). Amplification was performed using a quantitative thermal cycler (Roche, Light cycler 96, Switzerland). PCR measurements were performed in a total volume of 20 μL, containing 1.0 μL of each primer, 10 μL of 2 × conc. SYBR Green I Master (Roche, Switzerland), 2 μL of cDNA, and 6 μL DEPC-water. The procedure of quantitative PCR was employed: 95°C for 2 min, followed by 45 cycles of 95°C for 10 s, 58°C for 10 s, and 72°C for 20 s. Standard curves were generated using six different dilutions (in triplicate) and the amplification efficiency was analyzed as follows: *E* = 10^(–1/Slope)^ − 1 (Jothikumar et al., 2006). In this study, the gene expression was presented as relative gene expression, which we used as the relative quantification method to analyze data from RT-qPCR experiment. Expression levels of target genes were calculated using the 2^–ΔΔCt^ method (Livak and Schmittgen, 2001).

**TABLE 3:** Real time PCR primer sequences for analysis of gene expression in liver of black seabream.

**Function classification**	**Gene names**	**Primers**
Glycolysis	*gk*	F: GAGCAGGTTATGCCCATTGT
		R: TGGAAGTGAATGCGAGTCAG
	*pk*	F: CCGTCCCTTTCACTAATCCA
		R: CCGTCCCTTTCACTAATCCA
Gluconeogenesis	*pepck*	F: GGACCTGGCACGGTACTAAA
		R:CACGGGAAAACTGCTACCAT
	*g6pc*	F: TCTTCTGTCTTCCCCTGACG
		R: TTCTGCTTCATCTGCTCGAC
Glucose	*glut2*	F: ACAGAGGAGCGGATCAAAGA
transporter		R: GCAATCACTCCTGCTTCCTC
Lipogenesis	*acc*α	F: CGAGATGTTTCGCAATGAAA
		R: AGCTCCACGTTTGCGTAGTT
	*fas*	F: AGTGGGGAGTTGTTGGACAG
		R: ACAGTCGGCTCAAAGGAGAA
Fatty acid	*cpt1*	F: GGCAGATCATGTTTGTGTGC
β-oxidation		R: CATCGCTTACTTCCACAGCA
	*acox1*	F: CTTCACCCCTACATGCACCT
		R: CACTGTTGGCCTAGCACTGA
Long chain	*fads2*	F: GGTGGGCATGTTCTTGATCT
PUFA		R: ACTGTGTTCGGTCCTTCACC
biosynthesis	*elovl5*	F: TCATCCCGTGATGCTTTACA
		R:CACAGGGCAAACTTTTGGAT
	β*-actin*	F:CAGGACTCCATACCGAGGAA
		R:TGCGTGACATCAAGGAGAAG

**gk*, glucokinase; *pk*, pyruvate kinase; *pepck*, phosphoenolpyruvate; *g6pc*, glucose-6-phosphatase; *glut2*, glucose transporter 2; *acc*α, acetyl-CoA carboxylase alpha; *fas*, fatty acid synthase; *cpt1*, carnitine palmitoyl transferase 1; *acox1*, acyl-CoA oxidase 1; *fads2*, fatty acyl desaturase 2; *elovl5*, elongase 5.*

### Calculations and Statistical Analysis

The parameters were calculated as follows:

Percent weight gain (PWG, %) = 100 × (W_*t*_−W_*i*_) / W_*i*_
Survival (%) = 100×(final amount of fish) / (initial amount of fish)
Specific growth rate (SGR, % day^−1^) = 100 × (*Ln* W_*t*_−Ln W_*i*_)/ *t*
Protein efficiency ratio (PER) = weight gain (g) / protein intake (g)
Condition factor (CF, g cm^−3^) = 100 × (body weight, g) / (body length, cm) 3
Hepatosomatic index (HSI, %) = 100 × (liver weight / whole body weight)
Viscerosomatic index (VSI, %) = 100 × (viscera weight, g) / (body weight g)
Feed conversion ratio (FCR) = feed intake (g, dry weight) / weight gain (g, wet weight)

**FIGURE 1:**
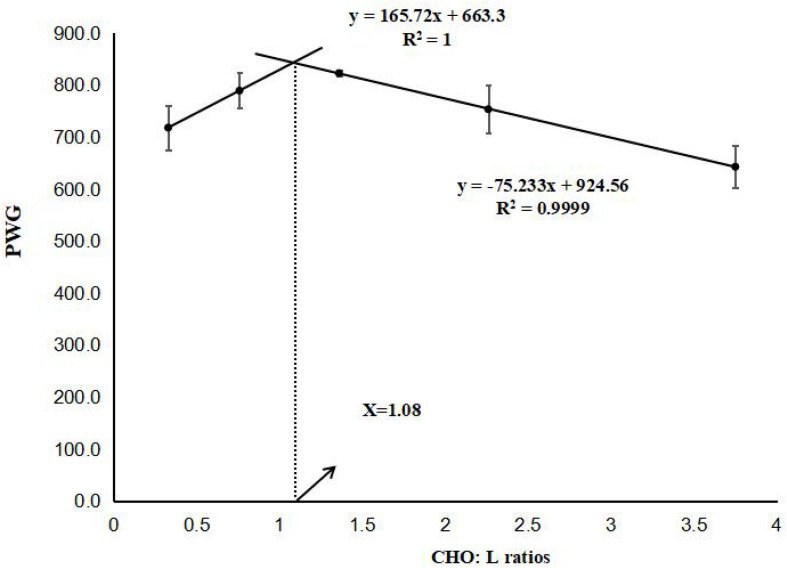
Relationship between percentage weight gain (PWG) and dietary (CHO:L) ratios based on two-slope, broken-line regression analysis, where Xopt represents the optimal dietary (CHO:L) ratio for the maximum PWG of black seabream.

Here, *W*_*t*_ is the final body weight (g), *W*_*i*_ is the initial body weight (g), *t* is the experimental duration in days. The results are presented as the means ± SE (*n* = 3). Prior to statistical analysis, normality and homogeneity of variance were checked, and percentage data were subjected to arcsine transformation. When the ANOVA identified significant differences between dietary treatments (*p* < 0.05), multiple comparisons were then made with Tukey’s test. A two-slope, broken-line regression analysis was conducted based on PWG to determine the optimum CHO:L ratio (Figure 1). All statistical analyses were performed using SPSS 23.0 (SPSS, IBM, United States).

## Results

### Growth Performance, Feed Utilization and Organosomatic Indices

The effects of different dietary CHO:L ratios on growth performance, feed utilization, and organosomatic indices are shown in Table 4. PWG, SGR, and PER were significantly influenced by dietary CHO:L ratio (*p* < 0.05). Fish fed the 1.36 CHO:L ratio diet had higher PWG, SGR, and PER than those fed the other diets. Two-slope, broken-line regression analysis of PWG against dietary CHO:L ratio indicated that the optimal dietary CHO:L ratio for juvenile black seabream is 1.08 (Figure 1). However, lower FCR was recorded in fish fed with 0.76, 1.36, and 2.26 CHO:L ratio diets than those fed the other diets. Survival ranged from 96 to 100%, and there was no significant difference among all dietary treatments (*p* > 0.05). HSI and VSI were significantly affected by the dietary CHO:L ratios (*p* < 0.05), but there were no significant differences observed in condition factor among all treatments (*p* > 0.05).

**TABLE 4:** Growth performance, feed utilization, and morphological indices of black seabream fed with experimental diets for 8 weeks.

**Parameters**	**Dietary carbohydrate to lipid ratios**					***p*-values**
	**0.33**	**0.76**	**1.36**	**2.26**	**3.75**	
IBW^1^ (g)	0.51 ± 0.02	0.51 ± 0.02	0.53 ± 0.01	0.52 ± 0.02	0.53 ± 0.01	0.632
FBW^2^ (g)	4.20 ± 0.25^bc^	4.53 ± 0.08^ab^	4.86 ± 0.05^a^	4.42 ± 0.40^ab^	3.91 ± 0.18^c^	0.005
PWG^3^ (%)	717.99 ± 43.17^bc^	789.25 ± 33.96^ab^	822.89 ± 5.27^a^	753.50 ± 45.76^ab^	642.83 ± 40.44^c^	0.001
SGR^4^ (% day^–1^)	3.75 ± 0.10^bc^	3.90 ± 0.07^ab^	3.97 ± 0.01^a^	3.83 ± 0.09^ab^	3.58 ± 0.10^c^	0.001
FCR^5^	1.37 ± 0.04^a^	1.28 ± 0.01^b^	1.27 ± 0.47^b^	1.27 ± 0.02^b^	1.36 ± 0.02^a^	0.001
PER^6^	1.55 ± 0.05^bc^	1.63 ± 0.05^a^	1.67 ± 0.00^a^	1.61 ± 0.04^ab^	1.52 ± 0.02^c^	0.001
Survival (%)	96.67 ± 5.77	100.00 ± 0.00	100.00 ± 0.00	100.00 ± 0.00	98.33 ± 2.89	0.552
HSI^7^ (%)	1.53 ± 0.45^b^	1.40 ± 0.26^b^	2.64 ± 0.28^a^	2.59 ± 0.25^a^	2.14 ± 0.27^a^	0.001
VSI^8^ (%)	7.73 ± 0.79^b^	6.26 ± 0.38^c^	8.62 ± 0.45^a^	6.90 ± 0.36^bc^	6.56 ± 0.86^c^	0.000
CF^9^ (g cm^–3^)	3.07 ± 0.19	3.30 ± 0.38	3.22 ± 0.24	3.37 ± 0.51	3.04 ± 0.42	0.770

*Values means ± SE (*n* = 3) in each row with different superscript letters are significantly different (*p* < 0.05). ^1^IBW, initial body weight; ^2^FBW, final body weight; ^3^PWG, percent weight gain; ^4^SGR, specific growth rate; ^5^FCR, feed conversion ratio; ^6^PER, protein efficiency ratio; ^7^HSI, hepatosomatic index; ^8^VSI, viscerosomatic index; ^9^CF, condition factor.*

**TABLE 5:** The whole-body composition of black seabream fed with experimental diets for 8 weeks.

**Parameters**	**Dietary carbohydrate to lipid ratios**					***p*-values**
	**0.33**	**0.76**	**1.36**	**2.26**	**3.75**	
Moisture (%)	72.71 ± 0.46	72.07 ± 0.43	72.19 ± 0.69	73.03 ± 0.22	72.63 ± 0.13	0.727
Protein (%)	16.30 ± 0.14	16.40 ± 0.24	16.32 ± 0.27	16.75 ± 0.26	16.60 ± 0.50	0.101
Lipid (%)	7.97 ± 0.83^a^	7.78 ± 0.81^a^	6.90 ± 0.34^ab^	6.61 ± 0.08^b^	4.96 ± 0.60^c^	0.001
Ash (%)	5.02 ± 0.59	5.02 ± 0.53	5.27 ± 0.19	5.45 ± 0.50	5.19 ± 0.35	0.732

*Values means ± SE (*n* = 3) in each row with different superscript letters are significantly different (*p* < 0.05).*

### Proximate Composition in the Whole Body

There were no significant differences in moisture, crude protein, and ash contents of the whole body among all treatments (*p* > 0.05). However, fish fed the 2.26 and 3.75 CHO:L diets had lower crude lipid content in the whole body than those fed the other diets (*p* < 0.05) (Table 5).

### Serum Biochemical Parameters

The effects of dietary CHO:L ratios on contents of serum glucose (GLU), cholesterol (CHOL), triglycerides (TG), and total protein (TP) are presented in Table 6. The TG and CHOL concentrations in serum were significantly affected by dietary CHO:L ratios (*p* < 0.05). CHOL in serum significantly decreased with the dietary CHO:L ratios increasing from 0.33 to 3.75. However, GLU and TP concentrations in serum had no statistical differences among all dietary treatments (*p* > 0.05). Hepatic glycogen concentration significantly increased with the dietary CHO:L ratios increasing from 0.33 to 3.75 (*p* < 0.05; Table 6).

**TABLE 6:** Hematological indices and liver glycogen content of black seabream fed with experimental diets for 8 weeks.

**Parameters**	**Dietary carbohydrate to lipid ratios**					***p*-values**
	**0.33**	**0.76**	**1.36**	**2.26**	**3.75**	
GLU^1^ (mmol/l)	2.63 ± 0.57	2.72 ± 0.17	3.08 ± 0.53	3.00 ± 0.44	2.66 ± 0.49	0.660
TG^2^ (mmol/l)	3.64 ± 0.31^a^	3.32 ± 0.33^a^	3.05 ± 0.46^a^	2.38 ± 0.23^b^	2.26 ± 0.40^b^	0.003
CHOL^3^ (mmol/l)	7.86 ± 0.10^a^	7.75 ± 0.33^a^	7.40 ± 0.25^a^	5.64 ± 0.81^b^	5.33 ± 0.05^b^	0.000
TP^4^ (g/l)	32.38 ± 0.69	32.79 ± 0.56	33.74 ± 1.31	31.98 ± 1.70	31.60 ± 1.66	0.351
GLG^5^ (mg/g)	19.21 ± 0.65^d^	21.72 ± 0.78^c^	21.36 ± 0.45^c^	25.71 ± 0.92^b^	27.85 ± 1.08^a^	0.000

*Values means ± SE (*n* = 3) in each row with different superscript letters are significantly different (*p* < 0.05). ^1^GLU, glucose; ^2^TG, triglycerides; ^3^CHOL, cholesterol; ^4^TP, total protein; ^5^GLG, liver glycogen.*

### Muscle Fatty Acid Composition

The fatty acid profiles (percentage of total fatty acids) of the muscle of black seabream fed different dietary CHO:L ratios are shown in Table 7. Overall, 19 fatty acids were observed and identified with the key fatty acids being palmitic acid (PA, C16:0), stearic acid (SA, C18:0), oleic acid (OA, C18:1n-9), linoleic acid (LA, C18:2n-6), eicosapentaenoic acid (EPA), and docosahexaenoic acid (DHA). Muscle fatty acid composition clearly reflected the dietary fatty acid composition. Significant differences were observed for most fatty acids in the muscle of black seabream fed the different dietary CHO:L ratios diets (*p* < 0.05). Fish fed the 1.36 CHO:L ratio diet showed significantly higher percentages of EPA, DHA, and n-3 PUFA in muscle than those fed the other diets (*p* < 0.05).

**TABLE 7:** Fatty acid composition (% of total fatty acid) of muscle of black seabream fed with experimental diets for 8 weeks.

**Parameters**	**Dietary carbohydrate to lipid ratios**					***p*-values**
	**0.33**	**0.76**	**1.36**	**2.26**	**3.75**	
C14:0	2.24 ± 0.31^a^	2.04 ± 0.05^ab^	2.03 ± 0.09^ab^	1.70 ± 0.20^b^	1.66 ± 0.15^b^	0.0015
C16:0	16.38 ± 0.64	16.41 ± 0.83	17.83 ± 1.34	17.84 ± 0.75	17.75 ± 0.58	0.125
C18:0	5.67 ± 0.04^b^	5.98 ± 0.72^b^	6.94 ± 0.53^ab^	7.49 ± 0.31^a^	7.54 ± 0.38^a^	0.002
C20:0	0.27 ± 0.04^ab^	0.29 ± 0.06^a^	0.23 ± 0.05^ab^	0.20 ± 0.03^bc^	0.19 ± 0.02^c^	0.040
SFA^1^	23.70 ± 0.69^b^	23.69 ± 2.25^b^	27.70 ± 1.07^a^	27.26 ± 0.99^a^	27.20 ± 1.13^a^	0.000
C16:1n	3.1 ± 0.38^a^	2.8 ± 0.07^ab^	2.69 ± 0.09^ab^	2.35 ± 0.23^b^	2.24 ± 0.20^b^	0.005
C18:1n-9	14.96 ± 1.24	14.13 ± 0.49	14.64 ± 1.03	13.53 ± 0.78	12.9 ± 0.83	0.109
C20:1n-9	1.23 ± 0.13^a^	1.11 ± 0.04^ab^	0.94 ± 0.02^bc^	0.94 ± 0.12^bc^	0.79 ± 0.04^c^	0.001
C22:1n-11	0.37 ± 0.03	0.37 ± 001	0.43 ± 0.01	0.42 ± 0.06	0.38 ± 0.03	0.083
MUFA^2^	19.66 ± 1.73^a^	18.41 ± 0.53^ab^	18.7 ± 1.10^ab^	17.24 ± 1.15^bc^	16.31 ± 1.09^c^	0.043
C18:2n-6	19.19 ± 1.21^a^	17.94 ± 0.47^ab^	16.61 ± 0.80^bc^	14.7 ± 1.02^c^	12.06 ± 0.54^d^	0.000
C18:3n-6	0.12 ± 0.04^b^	0.11 ± 0.04^b^	1.64 ± 0.09^a^	0.28 ± 0.38^b^	0.1 ± 0.01^b^	0.000
C20:2n-6	0.36 ± 0.04	0.43 ± 0.07	0.41 ± 0.02	0.36 ± 0.06	0.77 ± 0.60	0.360
C20:4n-6	0.95 ± 0.05^ab^	1.03 ± 0.09^a^	0.89 ± 0.01^ab^	0.98 ± 0.09^ab^	0.83 ± 0.02^b^	0.016
C22:4n-6	0.13 ± 0.05	0.15 ± 0.03	0.17 ± 0.03	0.18 ± 0.04	0.19 ± 0.00	0.324
n-6 PUFA^3^	20.74 ± 1.14^a^	19.66 ± 0.49^a^	19.73 ± 0.85^a^	16.49 ± 0.77^b^	13.95 ± 1.08^c^	0.000
C18:3n-3	1.92 ± 0.21^a^	1.78 ± 0.06^a^	1.64 ± 0.09^ab^	1.41 ± 0.08^bc^	1.15 ± 0.05^c^	0.000
C18:4n-3	0.67 ± 0.12^a^	0.56 ± 0.07^ab^	0.66 ± 0.07^a^	0.54 ± 0.09^ab^	0.46 ± 0.03^b^	0.055
C20:4n-3	0.41 ± 0.05^d^	0.43 ± 0.03^cd^	0.52 ± 0.01^bc^	0.56 ± 0.06^ab^	0.65 ± 0.04^a^	0.000
C20:5n-3 (EPA)	3.95 ± 0.16^b^	4.21 ± 0.25^ab^	4.50 ± 0.29^a^	4.29 ± 0.21^ab^	4.09 ± 0.08^ab^	0.081
C22:5n-3 (DPA)	1.32 ± 0.06^b^	1.49 ± 0.09^ab^	1.58 ± 0.02^a^	1.58 ± 0.09^a^	1.62 ± 0.10^a^	0.006
C22:6n-3 (DHA)	12.22 ± 0.69^bc^	12.91 ± 0.98^ab^	14.06 ± 0.52^a^	13.88 ± 0.59^a^	10.95 ± 0.67^c^	0.002
n-3 PUFA^4^	20.16 ± 0.66^c^	21.72 ± 0.90^ab^	22.52 ± 0.62^a^	22.15 ± 0.83^a^	20.59 ± 0.88^bc^	0.017
EPA + DHA	16.17 ± 0.70^b^	17.12 ± 1.23^ab^	17.35 ± 1.00^ab^	18.39 ± 0.79^a^	17.04 ± 0.75^ab^	0.003

*Data are presented as means ± SE (*n* = 3). Values in the same row with different superscripts are significantly different (*p* < 0.05). Some fatty acids, found in only trace amounts or not detected, such as C8:0, C12:0, C13:0, C15:0, C14:1n-7, and C20:5n-6 were not listed in Table 7. ^1^SFA, saturated fatty acids, ^2^MUFA, mono-unsaturated fatty acids; ^3^n-6 PUFA; n-6 polyunsaturated fatty acids; ^4^n-3 PUFA; n-3 polyunsaturated fatty acids.*

### Relative Expression of Lipid and Glucose Metabolism-Related Genes in Liver

**FIGURE 2:**
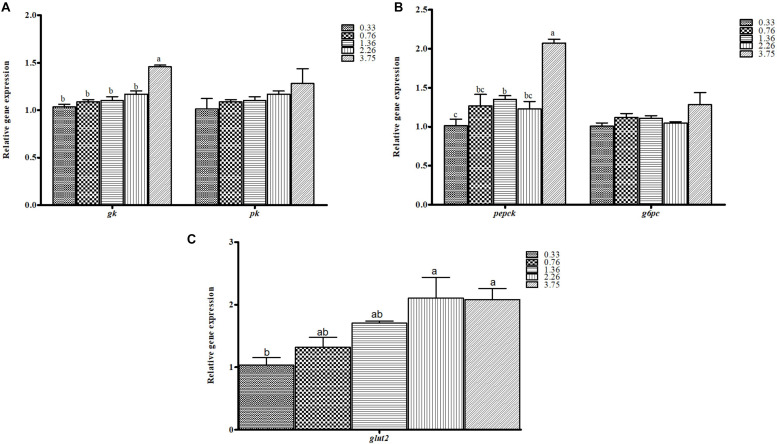
The mRNA expression levels of genes involved in **(A)** glycolysis (*gk*, glucokinase; *pk*, pyruvate kinase), **(B)** gluconeogenesis (*pepck*, phosphoenolpyruvate; *g6pc*, glucose-6-phosphatase), and **(C)** glucose transport (*glut2*, glucose transporter 2) in the liver of black seabream fed the different experimental diets. Expression values are normalized by β*-actin*. Data are expressed as means ± SE (*n* = 3). Values with different superscripts are significantly different (*p* < 0.05; Tukey’s range test).

The relative gene expression of glucose metabolism pathways in the liver of black seabream, including glycolysis (A), gluconeogenesis (B), and glucose transport (C), are shown in Figure 2. The expression level of glucokinase (*gk*), the key glycolytic enzyme, and of phosphoenolpyruvate carboxykinase (*pepck*), the key gluconeogenic enzyme, were significantly affected by dietary CHO:L ratios (*p* < 0.05). However, pyruvate kinase (*pk*) and glucose 6-phosphatase (*g6pc*), enzymes involved in the final step of glycolysis and gluconeogenesis, were not significantly influenced by dietary CHO:L ratios. The mRNA expression level of *pepck* was higher in fish fed with the carbohydrate-rich diet (*p* < 0.05). Moreover, the relative expression of *gk* significantly increased with increase of dietary CHO:L ratios. However, fish fed the 0.33 CHO:L diet had lower relative expression of *glut2*, which is involved in glucose transport, than those fed the 2.26 and 3.75 CHO:L ratio diets (*p* < 0.05).

**FIGURE 3:**
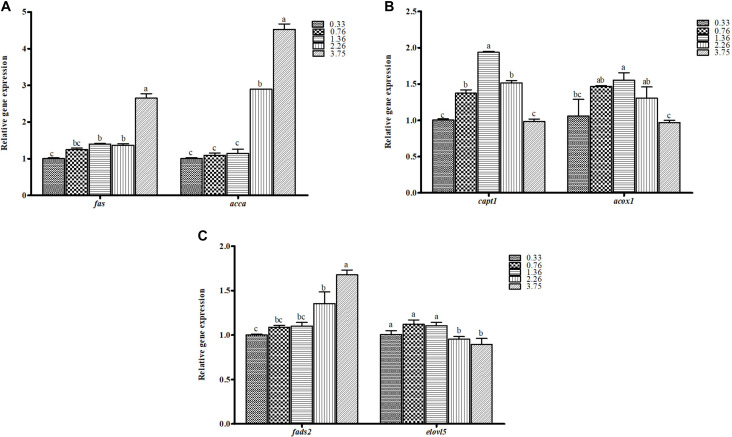
The mRNA expression levels of genes involved in **(A)** lipid synthesis (*fas*, fatty acid synthase; *acc*α, acetyl-coA carboxylase alpha), **(B)** fatty acid β-oxidation (*cpt1*, carnitine palmitoyl transferase 1; *acox1*, acyl-CoA oxidase), and **(C)** long-chain PUFA biosynthesis (*fads2*, fatty acyl desaturase; *elovl5*, elongase 5) in the liver of black seabream fed the different experimental diets. Expression values are normalized by β*-actin*. Data are expressed as means ± SE (*n* = 3). Values with different superscripts are significantly different (*p* < 0.05; Tukey’s range test).

The relative gene expressions involved in lipid biosynthesis in the liver of juvenile black seabream are shown in Figure 3. The higher mRNA expression level of lipogenic genes (*fas* and *acc*α) and lower mRNA expression of fatty acid β-oxidation genes (*cpt1* and *acox1*) were observed in fish fed with CHO:L of 1.36, 2.26, and 3.75 diets (*p* < 0.05). Fish fed the 1.36 CHO:L ratio had the highest expression of *cpt1* and *acox1* among all treatments. The mRNA expression levels of genes encoding key proteins involved in the LC-PUFA biosynthesis pathway (*elovl5* and *fads2*) were upregulated in the liver of black seabream (*p* < 0.05). However, the relative expression of *fads2* was significantly upregulated in fish fed the 3.75 CHO:L ratio diet.

## Discussion

The efficiency of protein can be enhanced through the utilization of carbohydrates and lipids in the diet for the cultured fish species (NRC, 2011). Various experimental studies have revealed that fish can efficiently utilize both carbohydrates and lipids to achieve better growth (Kim and Lee, 2005; Li et al., 2012a, b; Darias et al., 2015). In this study, the results demonstrated that PWG and SGR were significantly improved with dietary CHO:L ratios increasing up to 1.36, after which a decreasing trend was observed. These findings are consistent with previous studies indicating that appropriate dietary CHO:L ratios considerably improved growth performance in African catfish (*Clarias gariepinus*; Ali and Jauncey, 2004), dorado (*Salminus brasiliensis*; Moro et al., 2015), large yellow croaker (Zhou et al., 2016), and Nile tilapia (*Oreochromis niloticus*; Xie et al., 2017). These results also indicated that excessive dietary CHO:L ratios directly caused the growth depression in black seabream similar to other fish species (Ali and Al-Asgah, 2001; Moro et al., 2015; Xie et al., 2017). Hence, excessive increase in the input of non-protein energy sources always has a negative impact on growth performance, which appears to be closely associated with poor feed consumption by fish. Although an appropriate ratio of dietary carbohydrate and lipid is required, dietary carbohydrate is used to improve the palatability of the diet and maximize growth. However, lipids are used to fulfill the requirements for essential fatty acid in fish (Ng and Romano, 2013). In the present study, the survival was higher than 96% without any significant difference among all the treatments, suggesting that black seabream can thrive on a vast range of CHO:L ratios to attain better growth results.

Consistently, the feed ingredients not only affect the growth parameters of fish but also the physiological conditions, such as tissue physiology and plasma biochemical factors (Tian et al., 2012). In this study, the plasma triglyceride and cholesterol levels significantly decreased with increase of dietary CHO:L ratios, which was consistent with findings of other fish species (Hu et al., 2007; Zhou et al., 2016). The most favorable reason can be ascribed to active endogenous lipid transport in fish (Wang et al., 2014). However, the effects of CHO:L ratios on blood indexes, especially glucose, are still unclear, and further study is required.

In addition, a significant decrease in the whole-body lipid content with increase of dietary CHO:L ratios was observed in this study. It can be inferred that the whole-body lipid content in general accord with a previous study reported by Hu et al. (2007) that stated comparable results in yellowfin seabream (*Sparus latus*). However, conflicting conclusions observed in cobia (*Rachycentron canadum*) and European sea bass (*Dicentrarchus labrax*) were defined (Moreira et al., 2008; Ren et al., 2011). The present results also provided the credible fact that high carbohydrate intake resulted in a significantly high amount of HSI in fish (Brauge et al., 1994; Zuo et al., 2013). The high content of HSI with increasing levels of dietary carbohydrate was in agreement with the higher hepatic glycogen content, and these results were in accordance with the previous results on juvenile cobia (Ren et al., 2011) and rainbow trout (*Oncorhynchus mykiss*; Kamalam et al., 2012). The results of the present study indicate that the higher dietary carbohydrate level can promote glycogenesis and lipogenesis in fish (Moreira et al., 2008).

In the liver, the excess amount of dietary glucose is transformed into glycogen or lipids or used for energy. The production of pyruvate by glycolysis is either oxidized for energy or directed into pathways for lipogenesis (Uyeda and Repa, 2006). The glucose transportation rate and glycolysis potential can be increased by higher absorptions of carbohydrates, such as the upregulation of the hepatic genes *glut2* and *gk* in black seabream. This is in agreement with results in gilthead sea bream (*Sparus aurata*) and rainbow trout (Panserat et al., 2000), where the activity of *gk* was intensely upregulated by a rich carbohydrate diet. Although the lack of transcriptional regulation of *pk* by dietary carbohydrates may probably be associated to a post-transcriptional mechanism (Enes et al., 2006), there was no transcriptional regulation of *g6pc*, and key gluconeogenic enzyme *pepck* was downregulated by dietary carbohydrates. Taking into account these outcomes, we suggest that carbohydrate catabolism is regulated at a nutritional level in this species. These analytical interpretations are in agreement with the results from European sea bass and gilthead sea bream (Enes et al., 2011), where the activity of *pepck* recommend that gluconeogenesis is partly regulated by dietary carbohydrates at the transcriptional level.

The lipid metabolism in the liver is a very complex process; hepatocytes not only import and export lipids via lipoprotein, but they also oxidize lipids through fatty acid oxidation or synthesize new lipid by *de novo* lipogenesis (Akie and Cooper, 2015). It is generally reported that *fas* is the significant lipogenic enzyme for the anabolic alteration of dietary carbohydrates to fatty acids (Chen et al., 2015), and *acc*α is reflected as an important enzyme in the synthesis of long-chain poly-unsaturated fatty acids (Qian et al., 2015; Castro et al., 2016). In the present study, both *fas* and *acc*α played a vital role in fatty acid biosynthesis. The expressions of *fas* and *acc*α genes were all upregulated in the fish fed with a higher CHO:L ratio diet, and the results are parallel with previous work in other fish species (Xiong et al., 2014; He et al., 2015). The significant difference of these outcomes may possibly be due to the elevation of *de novo* lipogenesis in response to the elevated level of carbohydrates in the low-lipid diet (NRC, 2011). The gene expression of *cpt1*, a marker of mitochondrial FA β-oxidation was downregulated in the liver of fish fed with higher dietary carbohydrate and lower lipid diets. However, in a number of studies, the expression of the *cpt1* gene was not nutritionally regulated (Kennedy et al., 2006; Morais et al., 2011; Kamalam et al., 2012). Furthermore, lipogenesis and FA β-oxidation are two different pathways generally regulated in opposite directions (Zheng et al., 2014; Bonacic et al., 2016). The downregulated expression of *acox1* and *cpt1* were potentially associated with increasing dietary CHO:L ratios from 2.26 to 3.75; similar results were reported for *Ctenopharyngodon idellus* (Li et al., 2016). The possible reason can be endorsed to the provision of digestible carbohydrates in diets that could spare the use of lipids as source of energy (Garcia-Meilan et al., 2014).

On the other hand, the high amount of ARA, EPA, and DHA in fish muscle can also affect the gene expression of *cpt1* and *acox1* because lipid accumulation mostly takes place when excess lipids that were consumed by fish could not be oxidized (Lu et al., 2014). Contrary to humans and other mammals, dietary excessive LC-PUFA in fish oil supplementation decreased lipogenesis and triglyceridaemia (Ikeda et al., 1998; Davidson, 2006; Harris et al., 2008), and such effects are not clear in fish. Many studies in fish showed that fish oil either depressed (Jordal et al., 2007), had no particular effects (Regost et al., 2003; Richard et al., 2006), or had contrary effects (Menoyo et al., 2004) on lipogenesis. In this study, upregulation of *fads2* and *elovl5* were noticed. The improved transcript levels of *fads2* and *elovl5* were also examined in rainbow trout (Seiliez et al., 2001; Kamalam et al., 2013) and European sea bass (Geay et al., 2010). The maximum *fads2* efficiency is considered to be regulated by the levels of substrate and product availability (Vagner and Santigosa, 2011). In addition, this regulation may explain the capacity of conversion of C_18_ PUFA into LC-PUFA at an appreciable rate in fish species. Furthermore, the amount of n-3 LC-PUFA, principally EPA and DHA, decreased in the muscle of fish fed a carbohydrate-rich diet. The reduced n-3 LC-PUFA content in fish fed a diet with higher CHO:L ratios could be interconnected to an increase in SFA derived by lipogenesis from carbohydrates as in earlier findings in other species, such as rainbow trout and European sea bass (Alvarez et al., 1998; Castro et al., 2015). This suggested that the CHO:L ratios might affect the tissue FA composition and also FA biosynthesis of fish differently according to fish species, feeding habits, dietary carbohydrate level, and lipid sources (Castro et al., 2016). A future study concerning the dietary CHO:L ratios effect on the FA biosynthesis of black seabream is needed to elucidate. However, the levels of n-3 LC-PUFA (EPA and DHA) in the muscle of black seabream fed a dietarily optimal CHO:L ratio contributed possible health benefits to fish consumers.

## Conclusion

In conclusion, based on two-slope, broken-line regression analysis, the optimal dietary CHO:L ratio is recommended to be 1.08 for juvenile black seabream. Dietary CHO:L ratio could influence the tissue fatty acid profile and the accumulation of glycogen in tissues. Moreover, dietary CHO:L ratios upregulated or downregulated relative expression levels of genes involved in glucose and lipid metabolism. The results of the present study could provide important insight for molecular studies on fish nutrition and sustainable aquaculture development of black seabream.

## Data Availability Statement

The raw data supporting the conclusions of this article will be made available by the authors, without undue reservation, to any qualified researcher.

## Ethics Statement

In the present study, all procedures complied with Chinese law pertaining to experimental animals. The protocol was approved by the Ethic-Scientific Committee for Experiments on Animals of Ningbo University.

## Author Contributions

ST formulated the research question, designed the study, carried out the study, analyzed the data and wrote the manuscript. MJ designed the study, assisted in the correction, and developed the questions. MI designed the study, assisted in the correction, and revised the manuscript. YY assisted in the correction. HA was involved in data analysis. QZ formulated the research question, designed the study, and revised the manuscript. All the authors approved the final version of the manuscript.

## Conflict of Interest

The authors declare that the research was conducted in the absence of any commercial or financial relationships that could be construed as a potential conflict of interest.

## Abbreviations

*acc* α: acetyl-CoA carboxylase alpha
*acox1*: acyl-CoA oxidase 1
*cpt1*: carnitine palmitoyl transferase 1
*elovl5*: elongase 5
*g6pc*: glucose-6-phosphatase
*glut2*: glucose transporter 2
*gk*: glucokinase
*fads2*: fatty acyl desaturase 2
*fas*: fatty acid synthase
*pepck*: phosphoenolpyruvate
*pk*: pyruvate kinase.
